# Left ventricular endocardial and epicardial strain changes with apical myocardial ischemia in an open‐chest porcine model

**DOI:** 10.14814/phy2.13042

**Published:** 2016-12-30

**Authors:** Kimberly Howard‐Quijano, Melissa McCabe, Alexander Cheng, Wei Zhou, Kentaro Yamakawa, Einat Mazor, Jennifer C. Scovotti, Aman Mahajan

**Affiliations:** ^1^ Department of Anesthesiology and Perioperative MedicineDavid Geffen School of MedicineUCLA Health SystemLos AngelesCalifornia

**Keywords:** Echocardiography, ischemia, left ventricular function, strain

## Abstract

Early detection of acute myocardial ischemia is critical to prevent permanent myocardial damage. The impact of apical ischemia on global left ventricular (LV) function can be difficult to characterize using traditional volume‐based echocardiography measures. Myocardial strain imaging is a sensitive, quantitative marker of myocardial deformation that can measure ventricular function. Recent advances allow layer‐specific measurement of endo‐ and epicardial strain, enhancing the ability to evaluate myocardial ischemia. This study investigates the effects of apical ischemia on LV function using epi‐ and endocardial strain. We hypothesize that myocardial strain will identify changes in regional and global myocardial function associated with focal apical ischemia as compared to ejection fraction (EF), and that longitudinal strain will be a better indicator of myocardial dysfunction compared to circumferential or radial strain. In a porcine model (*n* = 9), acute ischemia was induced by left anterior descending coronary artery occlusion. Echocardiograms were performed at baseline, during 15‐min ischemia, and after reperfusion. Global longitudinal strain decreased with acute focal ischemia of the left ventricular apical region (baseline: −16.4% vs. ischemia: −12.2%; *P* = 0.010), with no change observed in global circumferential and radial strain or EF. Both endocardial and epicardial longitudinal strain decreased by 68% (*P* < 0.001) in the ischemic and peri‐ischemic zone, while circumferential and radial strain only decreased in endocardium of the ischemic zone. Longitudinal strain was more sensitive to ischemia, being able to detect changes in global LV function and thus may confer clinical diagnostic advantage in the evaluation of acute LV apical ischemia.

## Introduction

Early detection of acute myocardial ischemia is critical for prevention of irreversible myocardial damage. Subtle changes in endocardial deformation can now be measured quantitatively with speckle tracking strain imaging (Mor‐Avi et al. 2011). Myocardial strain is a sensitive, quantitative marker of myocardial deformation (Voigt et al. 2003); strain measured by speckle tracking echocardiography is angle‐independent and has been validated by sonomicrometry and cardiac MRI (Korinek et al. 2005; Langeland et al. 2005; Amundsen et al. 2006; Altiok et al. 2013; Koos et al. 2013). Myocardial strain has been found to improve detection of ischemia‐induced regional wall motion abnormalities in stress echocardiography studies as compared to qualitative echocardiography assessment alone (Reant et al. 2008). In addition, global longitudinal strain has been shown to be a strong clinical predictor of morbidity and mortality associated with myocardial dysfunction (Ternacle et al. 2013a).

New developments in speckle tracking echocardiography allow layer‐specific measurement of strain with the ability to differentiate epicardial from endocardial deformation. While conventional myocardial strain measurements have been shown to detect myocardial ischemia reliably, they are taken at the interface of the epicardial and endocardial layers and are influenced by layer interdependence (Altiok et al. 2013). Layer‐specific strain has been shown to be a more sensitive marker of nontransmural chronic infarction than conventional myocardial strain (Becker et al. 2009). Given the findings in chronic ischemia, layer‐specific strain may be useful in studying the effects of acute ischemia, where layer‐specific differences may be present as ischemia progresses from the endocardium to the epicardium.

Apical ischemia is commonly seen in clinical practice, especially after cardiac surgery (Swaminathan et al. 2007; Zhou et al. 2013). The impact of apical ischemia on global left ventricular (LV) function can be difficult to characterize using volume‐based measures such as ejection fraction (EF) given the small contribution of the apex to global LV volume and mass. The effect of isolated apical ischemia on global LV function has not been well characterized. Given the complex anatomical structure and orientation of myocardial fibers, acute focal ischemia may have different effects on longitudinal versus circumferential and radial strain (Greenbaum et al. 1981; Jones et al. 1990). This study investigates the effects of acute apical ischemia on global and regional LV function using layer‐specific epi‐ and endocardial longitudinal, circumferential, and radial strains. We hypothesize that myocardial strain will identify changes in regional and global myocardial function associated with focal apical ischemia as compared to EF. In addition, since the longitudinal myocardial fibers contribute significantly to the apical region and run along length of the LV, we hypothesize that longitudinal strain will be a better indicator of myocardial dysfunction compared to circumferential or radial strain.

## Methods

### Animal preparation

**Figure 1:**
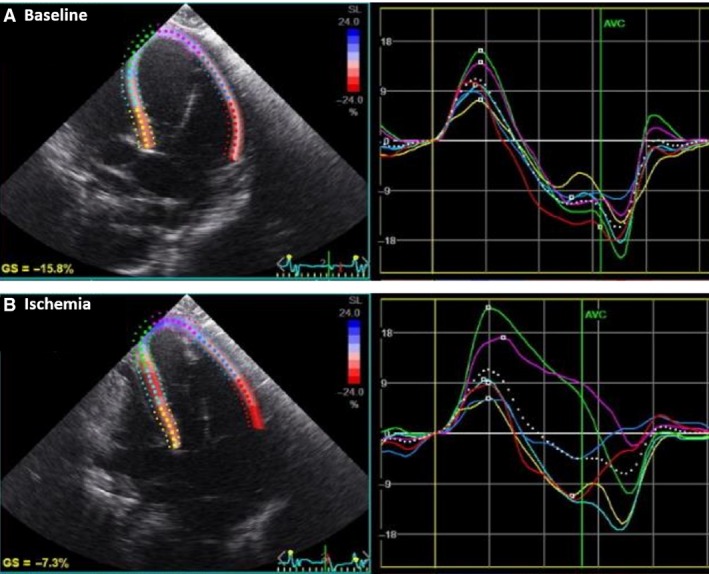
Global longitudinal strain (LS). Representative 4‐chamber view of the left ventricle. (A) Peak systolic LS at baseline and (B) After 15‐min ischemia, demonstrating regional apical dysfunction and a reduction in global LS.

**Figure 2:**
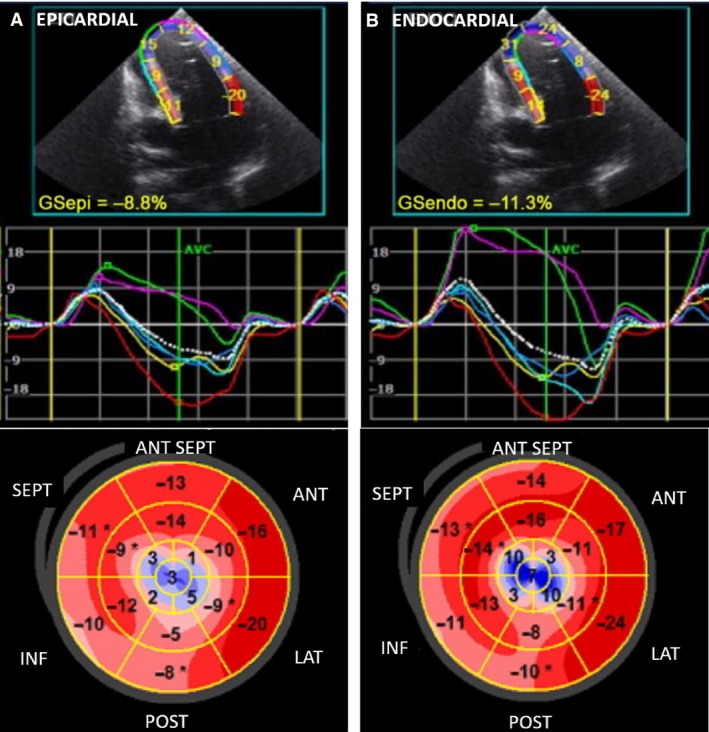
Layer‐specific longitudinal strain (LS). (A) Representative epicardial and endocardial peak systolic LS of the 4‐chamber view of the left ventricle (LV) and (B) Representative epicardial and endocardial bullseye diagram of the LV segmental anatomy demonstrating left ventricular apical ischemia represented by systolic dyskinesis in blue. ANT SEPT = anterior septal LV wall, ANT = anterior, LAT = lateral, POST = posterior, INF = inferior, and SEPT = septal wall.

This study was approved by the Chancellor's Animal Research Committee of the University of California (Los Angeles, CA) and complied with National Institutes of Health guidelines on the care and use of laboratory animals. Yorkshire pigs (*n* = 9) weighing 40–50 kg were anesthetized intramuscularly with telazol (8–10 mg/kg), intubated, and mechanically ventilated. General anesthesia was maintained with isoflurane (1–2%). The electrocardiogram was monitored from limb and precordial leads. A femoral artery was cannulated for blood pressure measurement and a jugular vein was cannulated for drug infusion. A median sternotomy exposed the heart; the heart was suspended in a pericardial cradle and the second diagonal branch of the left anterior descending artery was isolated. After a 30‐minute equilibration phase, the second diagonal branch of the left anterior descending coronary artery was occluded with a 4.0 silk suture for 15 minutes followed by 30 minutes of reperfusion, with reproducible apical ischemia (Figs. 1, 2).

### Pressure‐volume measurements by conductance catheter

A 5‐French Millar conductance pressure‐volume catheter (Millar Instruments, Houston TX) was inserted via the left common carotid artery into the left ventricular, with echocardiography confirmation, and connected to a conductance processor (MPVS Ultra, Millar Instruments) to continuously measure left ventricular pressure and volume. Segmental volume signals were examined to ensure proper electrode position. Measures were acquired from steady‐state pressure‐volume loops during sinus rhythm. Left ventricular systolic function was assessed by stroke work (SW) and the maximum rate of pressure change (dP/d*t*_max_), while measures of left ventricular diastolic function included the minimum rate of left ventricular pressure change (dP/d*t*_min_) and the isovolumetric relaxation time constant (tau).

### Echocardiography

Open chest, epicardial echocardiography was performed according to the American Society of Echocardiography Guidelines for Comprehensive Epicardial Echocardiography Examination using GE Vivid 7 Dimension ultrasound system equipped with a 10 MHz phased‐array transducer (GE Vingmed, Horton, Norway). A bovine liver tissue offset was used to enhance image quality; all images were optimized for speckle tracking analysis by manually adjusting sector widths to optimize speckle quality and maintaining frame rates 55–90 frames/sec. Echocardiographic and hemodynamic data were acquired simultaneously at baseline and 1, 5, and 15 minutes after induction of ischemia. Measurements were repeated 1, 15, and 30 minutes after reperfusion. All images were obtained by the same echocardiographer (EM).

### Two‐dimensional speckle tracking strain

Strain analysis was completed offline using speckle tracking software (EchoPac PC Clinical Workstation v.113, GE Vingmed Ultrasound, Horton, Norway). All measurements were obtained using the average of three cardiac cycles and recorded for subsequent offline analysis. Apical and mitral annular points were identified in end‐diastolic and end‐systolic frames. The software's automated border tracking algorithm automatically defined the contour of epicardial and endocardial layers. The integrity of automated tracking was confirmed by inspection of speckle tracking and strain curves; if needed, the region of interest was adjusted manually to optimize tracking (performed in 4% of analyses). Segments with persistently inadequate tracking were excluded from the analysis.

Peak systolic longitudinal (LS) global and segmental strain was measured from the apical window using 4‐chamber, 2‐chamber, and long‐axis images. Representative 4‐chamber strain tracing demonstrating global LS output is shown in Figure 1 and representative epicardial and endocardial peak systolic LS with apical ischemia is demonstrated in blue in Figure 2. Circumferential (CS) global and segmental strain was measured in short axis at basal (identified by mitral valve), midventricular (midpapillary), and apical levels (subpapillary, prior to cavity obliteration). Radial strain (RS) was measured at basal (identified by mitral valve) and apical levels (subpapillary, prior to cavity obliteration) from the same short‐axis views.

The myocardial segments of the left ventricle were divided into three zones a priori based on direct visualization of myocardial tissue injury during occlusion of the second diagonal branch of the left anterior descending artery (LAD): nonischemic, ischemic, and peri‐ischemic. The ischemic zone was defined as the area of myocardial ischemia, and the peri‐ischemic zone contained the surrounding two centimeters of myocardium in LAD territory. The nonischemic zone included segments greater than two centimeters removed from ischemia.

### Statistical analysis

Hemodynamics, Millar catheter measurements, and myocardial strain measures are reported as means with standard deviation at each condition. The change in myocardial strain between each condition and baseline are reported as mean change expressed as percentage. Global myocardial strain measured at 15‐minute ischemia was compared to baseline using a paired t‐test. Hemodynamics and myocardial strain measures during ischemia and reperfusion were compared to baseline within ischemic, peri‐ischemic, and nonischemic zones using repeated measures ANOVA and Tukey's HSD stratified by myocardial layer (epicardial vs. endocardial). For zones with significant changes from baseline during ischemia, segmental analysis was performed using paired t‐tests to identify segments affected by ischemia. Bonferroni correction was used to account for multiple hypothesis testing and *P* < 0.05 was considered statistically significant. All statistical analyses were performed in JMP Pro v12.0 (SAS, Cary, North Carolina).

## Results

All nine animals completed study protocol with adequate images for speckle tracking strain analysis. Two animals experienced ventricular tachycardia (VT) at <1 min reperfusion. VT was nonsustained, no treatment was necessary, and all strain measurements were able to be analyzed at each time point.

### Baseline echocardiography and strain measures

**Figure 3:**
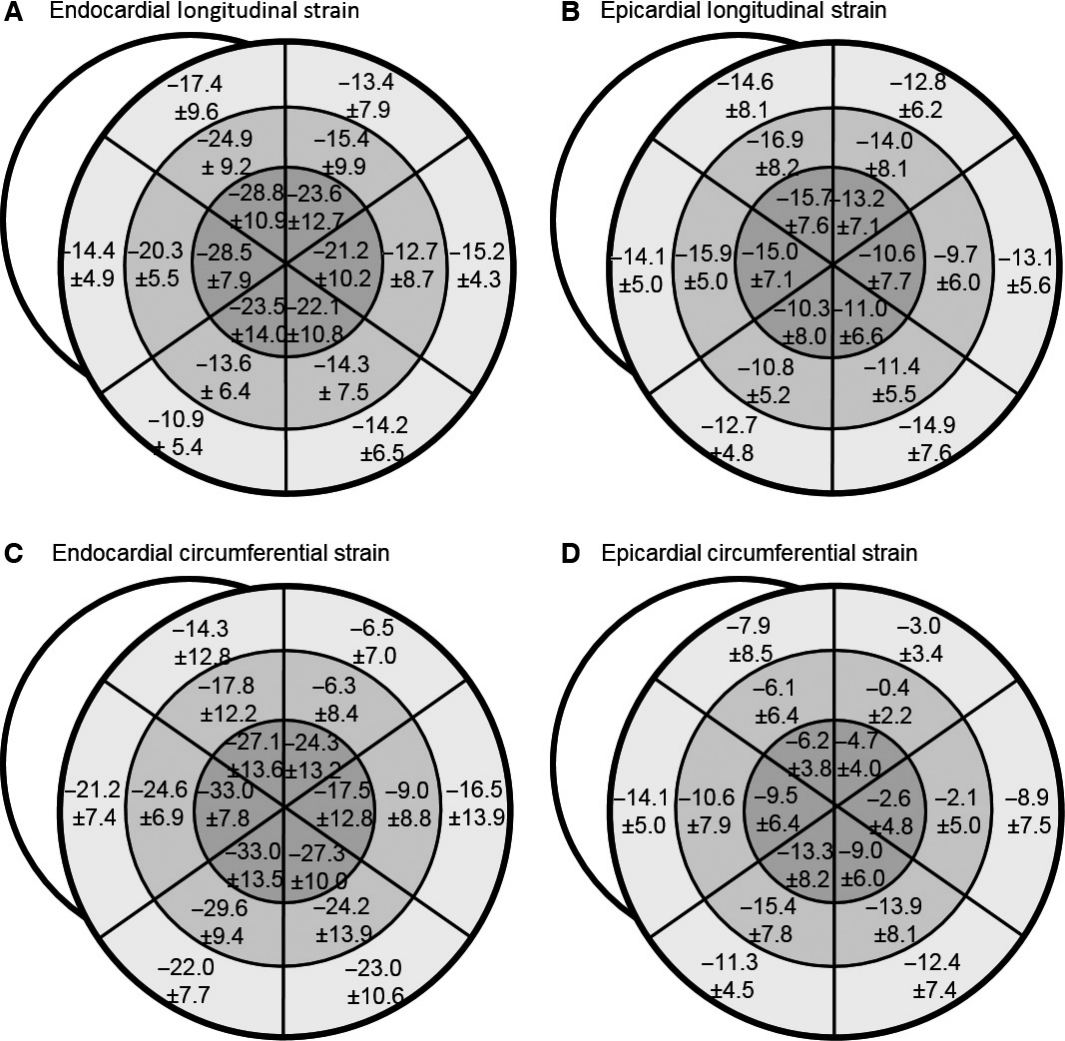
Baseline myocardial strain values. Segmental myocardial strain is displayed as mean ± SD. Baseline longitudinal and circumferential strain was greater at the apex than the base and endocardial strain was greater than epicardial strain.

All animals had normal two‐dimensional echocardiograms, and measurements of global longitudinal (LS), radial (RS), and circumferential (CS) strains were taken at baseline (Fig. 2). Regionally, endocardial strain was found to be greater than epicardial strain in both LS (−18.6 vs. −13.2, *P* < 0.001) and CS (−20.8 vs. −8.0, *P* = 0.0001) measures. Endocardial LS also had a baseline gradient of increasing values from base to mid (−14.3 vs. −17.0, *P* = 0.026) and mid to apex (−17.0 vs. −24.6, *P* = 0.003, Fig. 3), with no significant gradient in epicardial strain.

### Long‐axis left ventricular function

**Figure 4:**
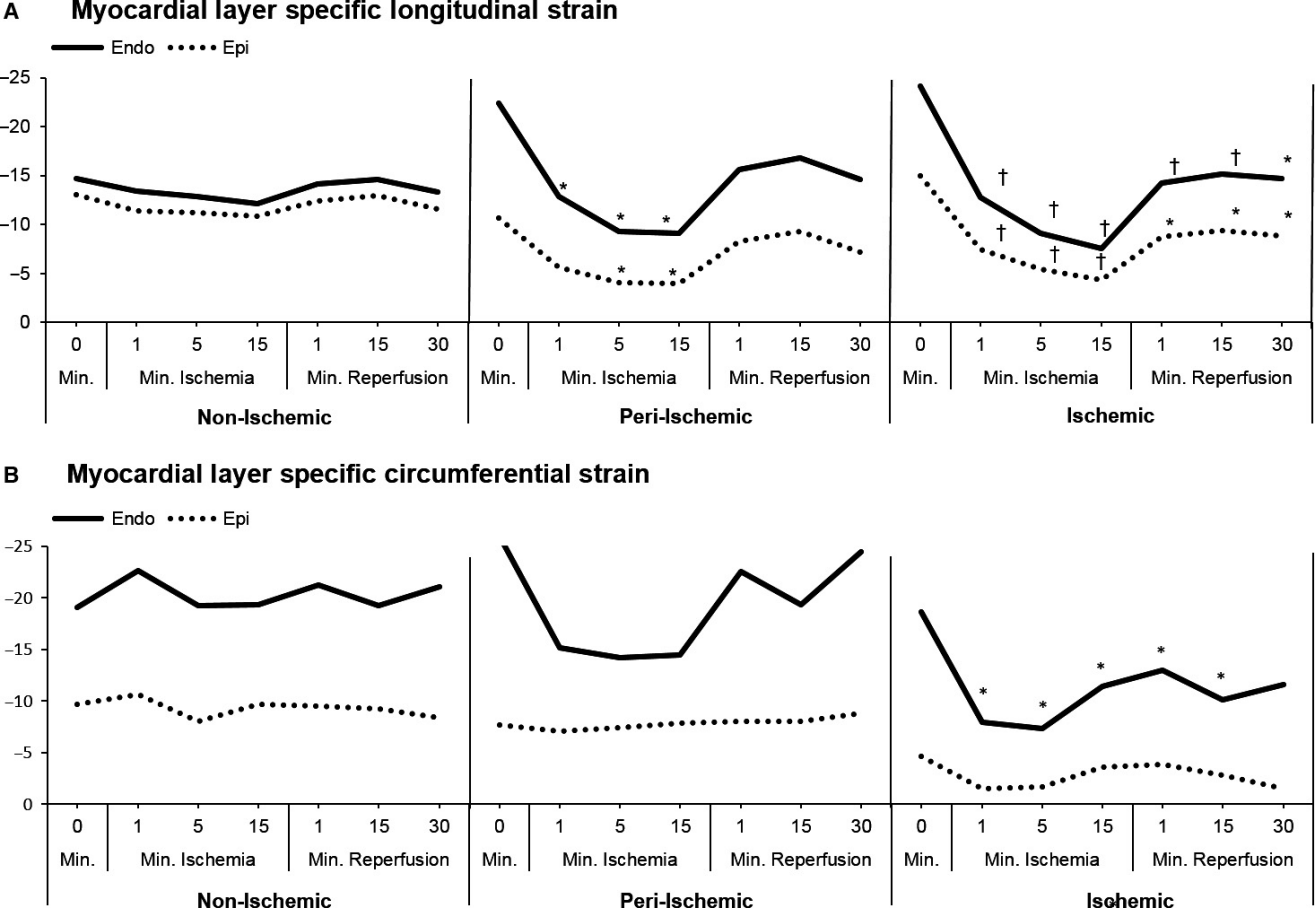
Myocardial layer‐specific segmental strain (A) Longitudinal strain (LS): Endocardial strain is greater than epicardial strain at all time points in the peri‐ischemic and ischemic zones (*P* < 0.04). LS decreased in the ischemic and peri‐ischemic zone significantly with ischemia. No significant change within the nonischemic zone. (B) Circumferential strain (CS): Endocardial strain is greater than epicardial strain at all time points (*P* < 0.001). CS decreased significantly only in the endocardium in the ischemic zone. *= *P* < 0.002 †=*P* ≤ 0.0001

Global LS decreased with acute ischemia of the left ventricular apex (Table 1). Regionally, LS decreased continuously within the ischemic zone with induction of ischemia, falling 46% after 1 minute, 63% after 5 minutes, and 68% below baseline after 15‐minute ischemia (*P* < 0.001, Fig. 4A). Segmental analysis of the ischemic zone during peak ischemia demonstrated decreases in both the endocardium and epicardium (*P* < 0.0001). Focal ischemia also affected the adjacent peri‐ischemic zone (Fig. 4A), where LS fell 39% and 32% below baseline after 15‐minute ischemia in the endocardium (*P* < 0.001) and epicardium (*P* < 0.038), respectively. Within the ischemic zone, partial recovery was present after 1‐minute reperfusion, but was attenuated thereafter, with LS remaining below baseline in the endocardium (*P* < 0.0001) and epicardium (*P* < 0.002) after 30‐minute reperfusion. LS did not change significantly in the nonischemic zone.

**Table 1:** Global myocardial function measures

	Baseline	15‐min ischemia	*P*‐value
Global strain			
Longitudinal	−16.4 ± 4.4	−12.2 ± 3.0	*P* = 0.010*
Circumferential	−12.7 ± 4.0	−11.9 ± 3.0	*P* = 0.557
Radial	41.7 ± 16.5	34.6 ± 10.9	*P* = 0.051
Ejection fraction	52.4 ± 9.5	45.7 ± 6.3	*P* = 0.658

Data expressed as mean ± standard deviation.

### Short‐axis left ventricular function

Global CS and RS were not significantly affected by acute ischemia (Table 1). Regionally, in the ischemic zone, CS was reduced by 45% in the endocardium at 1‐minute ischemia (*P* < 0.009). The nadir in CS occurred at 5‐minute ischemia and in the endocardium CS was 53% below baseline (*P* < 0.001) with no change in the epicardium (Fig. 4B). CS recovery preceded reperfusion. CS was not significantly decreased in the peri‐ischemic or nonischemic zones. RS followed a similar pattern to CS in which strain was reduced only in the endocardium of the ischemic zone (*P* < 0.03), with no other significant changes observed.

**Figure 5:**
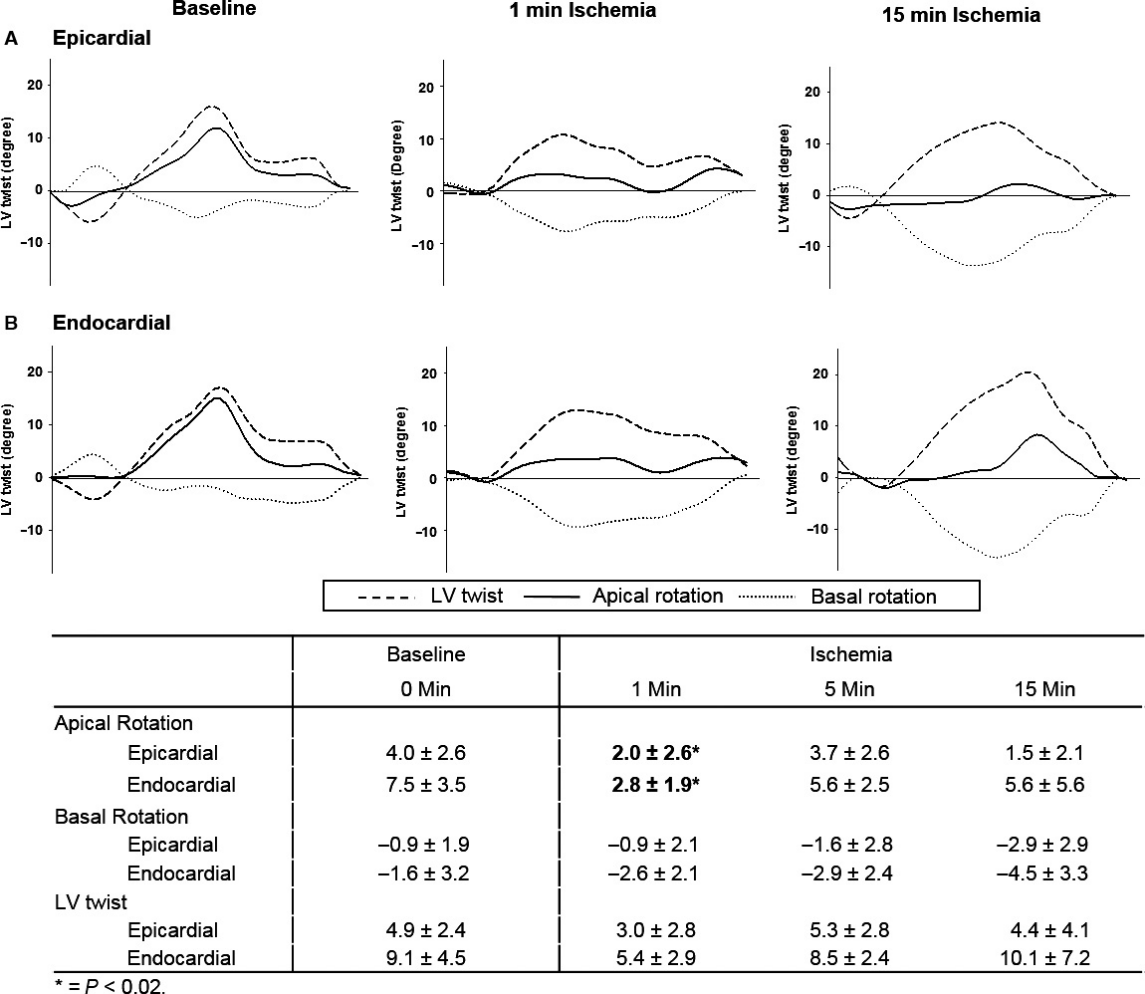
Representative example of left ventricular (LV) rotation and twist changes with transient apical ischemia. (A) Epicardial and (B) endocardial apical rotation, basal rotation, and LV twist at baseline and after 1‐ and 15‐minute ischemia. Apical rotation was significantly reduced at 1‐min ischemia, * = *P* < 0.02.

Apical rotation in both the endocardial and epicardial layers decreased significantly with acute ischemia, reaching its lowest value at 1‐minute ischemia (*P* = 0.02) before recovering partially at 5‐ and 15‐min ischemia (Fig. 5). Basal apical rotation in the epicardial and endocardial layers was not significantly affected by acute ischemia. Left ventricular twist also did not change significantly through ischemia and reperfusion.

### Hemodynamics and ejection fraction

**Figure 6:**
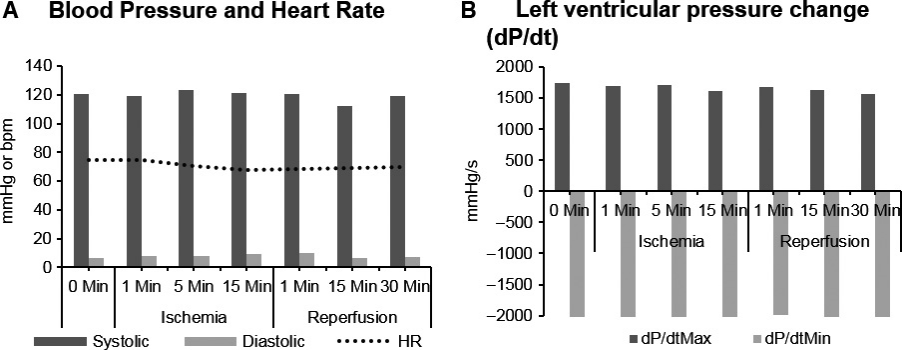
Hemodynamic changes with ischemia and reperfusion. (A) Blood pressure and heart rate. (B) Left ventricular pressure change (dP/dt minimum and maximum).

Blood pressure, heart rate, rate of left ventricular pressure change, and the isovolumic relaxation constant did not change significantly during ischemia or reperfusion (Fig. 6). There was no significant change in left ventricular stroke work throughout ischemia and reperfusion (mean ± SD); baseline 1264 ± 314, ischemia at 1 min 1299 ± 282, 5 min 1112 ± 380, 15 min 1346 ± 409 and reperfusion at 1 min 1103 ± 500, 15 min 1197 ± 262, and 30 min 1165 ± 351 mmHgml. There was also no change in ejection fraction value from baseline with ischemia (Table 1).

## Discussion

The results of this study show that myocardial strain measures are able to detect changes in global LV function during apical ischemia. Major findings include: (1) Global LS was reduced with acute LV apical ischemia, whereas global CS, RS, and EF were not; (2) In the ischemic zone, LS was reduced throughout ischemia in both the endocardium and epicardium, whereas CS and RS were reduced only transiently in the endocardium; (3) In the peri‐ischemic zone, endocardial and epicardial LS were both depressed while no changes in CS or RS were observed; (4) Apical rotation was also reduced transiently at 1‐min ischemia and then recovered. These results demonstrate that acute LV apical ischemia has a greater effect on LS, as compared to other measures of LV function, and therefore global LS may be a better clinical measure of LV ischemic apical dysfunction.

### Global and regional differences in longitudinal and circumferential strain

In this study, we have importantly shown that acute regional apical ischemia has differential effects on global long‐ and short‐axis myocardial function of the left ventricle. During LV, apical ischemia LS was affected to a greater degree than CS or RS. Traditionally, global LV ventricular function has been most commonly measured by EF, which uses changes in volume to indirectly determine ventricular function. Recent technological advancements in speckle tracking imaging allow direct measurement of myocardial deformation or strain (Amundsen et al. 2006). Peak systolic longitudinal strain measures myocardial function in the LV long axis, whereas circumferential and radial strain measure function in the short axis. In this study, we have demonstrated that global LS was reduced during focal LV apical ischemia, however, CS, RS, and EF were not. The significant reduction in both endo‐ and epicardial regional LS led to the observed decrement in global LS, whereas conversely, regional CS and RS were only transiently reduced in the endocardial layer. In the peri‐ischemic zone, LS was depressed while CS remained unchanged. Though strain measures and apical rotation were all initially reduced at 1‐minute ischemia, CS and apical rotation recovered, while LS declined progressively with ischemia.

The increased sensitivity of LS to apical ischemia may be due to the complex anatomical structure and functional mechanics of the LV which cause; (1) layer‐specific changes in fiber orientation and coronary perfusion predisposing endocardial and longitudinal fibers to ischemia. Circumferential fibers, which predominate in the midwall of the myocardium, become less concentrated toward the LV apex (Bogaert and Rademakers 2001). As a greater proportion of longitudinal fibers lie at the apex, the ratio of circumferential to longitudinal fibers is decreased (Leitman et al. 2010). During acute apical ischemia, longitudinal fibers are most affected with decreases in both endocardial and epicardial strain, whereas only endocardial circumferential strain is reduced with apical ischemia, As well as (2) complex coupling between different strains whereby CS compensates for reductions in LS (Mizuguchi et al. 2008; Carasso et al. 2013), and (3) morphologically, longitudinal fibers are arranged along the length of the LV from the apex to base; accordingly, apical ischemia impacting longitudinal fibers may result in larger effects on global LS. Although the diameter of the LV is smallest at the apex, so decreases in apical CS have a lesser contribution to global measures of CS (Voigt et al. 2003).

Previous studies in both clinical and experimental models support our observed results; they show that LS is affected earliest in subendocardial infarction and is a more sensitive marker after acute myocardial infarction as compared to CS or RS (Chan et al. 2006; Carasso et al. 2013; Altiok et al. 2014). Ternacle et al., investigated the changes in strain in patients with focal ischemia undergoing percutaneous coronary interventions and reported findings similar to our animal model in that while EF was unchanged during acute LAD occlusion, 2D LS was reduced (Ternacle et al. 2013b). However, Ternacle did not evaluate 2D CS, RS, or differential epi‐ and endocardial strain during acute ischemia. In contrast to the results, we describe with isolated LV apical ischemia in which CS was only reduced in the endocardium; studies involving diffuse anterior wall ischemia, have found reductions in regional midwall CS, however, the effect of regional ischemia on global LV function measures were not assessed in these studies.(Moen et al. 2011; Bachner‐Hinenzon et al. 2016).

### Clinical implications

The differences demonstrated in both global and regional strain measures during apical ischemia have important clinical implications. Left anterior descending coronary artery disease is the most common pathology encountered clinically and can lead to LV apical dysfunction (Altiok et al. 2014). Additionally, apical ischemia can occur after routine cardiac surgery impacting clinical outcomes. Myocardial dysfunction is most commonly assessed qualitatively via echocardiography using visual wall motion scores (WMS) by analyzing short‐axis images of the LV for changes in radial thickening and circumferential shortening (Carasso et al. 2013). As such, differences in the effect of focal ischemia on short‐ and long‐axis ventricular function will likely have notable diagnostic implications (Carasso et al. 2013).

Taken together, these findings suggest the use of global longitudinal strain in the evaluation of early LV apical ischemia. Changes in global LS may precede changes in hemodynamics or other ventricular function measurements. In our study population of healthy animals, without evidence of prior myocardial dysfunction, induction of acute LV apical ischemia caused no significant changes in hemodynamics or EF. Yet, significant changes were seen in global longitudinal strain, while CS and RS remained unchanged. The importance of LS as a predictor of outcomes, in comparison with other measures of LV function, has been previously established in several surgical populations. Baseline global LS has been shown to be an independent predictor of long‐term LV dysfunction in patients following mitral valve repair (Witkowski et al. 2013). Global LS was also shown to have incremental value beyond EF in predicting mortality and inotropic support in patients undergoing coronary artery bypass or left heart valve surgery (Ternacle et al. 2013a). In the dynamic intraoperative period, when changes in hemodynamics or EF may not be readily appreciated, global LS may be a superior measure to that of global CS or RS in detecting early LV apical ischemia and future studies will be needed to look at the utility of this emerging technology in the operative setting.

### Limitations

As this study used an animal model without preexisting coronary artery disease in order to see the specific effects of acute ischemia on myocardial function, the generalizability of these results may be limited in subjects with preexisting coronary artery disease and myocardial dysfunction. The majority of previous studies investigating the layer‐specific strain have been performed in chronic infarction models. Therefore, our study is unique in that we are applying this technology to an acute ischemia model. While we did not compare strain to another reference such as MRI in this study, layer‐specific strain has been validated by invasive sonomicrometry during acute ischemia (Langeland et al. 2005). In addition, inhaled isoflurane was used as the primary anesthetic in this study and may have cardioprotective effects during ischemia, thus underestimating the effects of ischemia on the myocardium.

## Conclusions

Myocardial strain measures are able to detect changes in global LV function during apical ischemia. LV apical ischemia had a greater effect on LS as compared to CS or RS and changes in global LS were demonstrated to be associated with LV apical dysfunction. Layer‐specific strain provides new insight to the changes in myocardial deformation associated with acute ischemia. The global strain findings in this study are likely directly related to the changes observed in regional myocardial function. LS may thus confer clinical diagnostic advantage in the evaluation of acute myocardial ischemia over traditional short‐axis analysis of wall motion abnormalities or EF.

## Conflict of Interest

No conflicts of interest, financial or otherwise, are declared by the authors.
